# Ethanol extract of propolis protects macrophages from oxidized low density lipoprotein-induced apoptosis by inhibiting CD36 expression and endoplasmic reticulum stress-C/EBP homologous protein pathway

**DOI:** 10.1186/s12906-015-0759-4

**Published:** 2015-07-14

**Authors:** Hua Tian, Hong-Wei Sun, Jia-Jun Zhang, Xiao-Wei Zhang, Li Zhao, Shou-Dong Guo, Yan-Yan Li, Peng Jiao, Hao Wang, Shu-Cun Qin, Shu-Tong Yao

**Affiliations:** Key Laboratory of Atherosclerosis in Universities of Shandong, Institute of Atherosclerosis, Taishan Medical University, Taian, 271000 China; Taishan Hospital of Shandong province, Taian, 271000 China; Affiliated Hospital of Taishan Medical University, Taishan Medical University, Taian, 271000 China; College of Basic Medical Sciences, Taishan Medical University, Taian, 271000 China

**Keywords:** Ethanol extract of propolis, Endoplasmic reticulum stress, C/EBP homologous protein, Oxidized low density lipoprotein, Macrophage, Apoptosis

## Abstract

**Background:**

Ethanol extract of propolis (EEP), rich in flavones, has been known for various biological activities including antioxidant, antiinflammatory and antibiotic activities. Our previous studies have shown that EEP protects endothelial cells from oxidized low-density lipoprotein (ox-LDL)-induced apoptosis and inhibits atherosclerotic lesion development. In this present study, we explored the protective effect of EEP on ox-LDL-induced cytotoxicity in macrophages and specifically the endoplasmic reticulum (ER) stress-C/EBP homologous protein (CHOP) pathway-mediated apoptosis.

**Methods:**

EEP was prepared and the total flavonoids content of EEP was determined by the colorimetric method of Chinese Standard (GB/T 20574-2006). The effects of EEP on lipid accumulation, cytotoxicity and apoptosis in RAW264.7 cells induced by ox-LDL or tunicamycin (TM, an ER stress inducer) were assayed using oil red O staining, MTT assay, flow cytometric analysis and so on. Immunofluorescence, Western blot and real time-PCR analysis were then used to further investigate the molecular mechanisms by which EEP protects macrophages from ox-LDL-induced apoptosis. 4-phenylbutyric acid (PBA), an ER stress inhibitor, was used as a positive control.

**Results:**

EEP (7.5, 15 and 30 mg/L) not only attenuated ox-LDL-induced lipid accumulation in RAW264.7 macrophages in a dose-dependent manner but also inhibited the decreased cell viability and the increased lactate dehydrogenase (LDH) leakage, caspase-3 activation and apoptosis induced by ox-LDL or tunicamycin (TM, a classical ER stress inducer), which were similar to 4-phenylbutyric acid (PBA, an inhibitor of ER stress) treatment. In addition, like PBA, EEP significantly suppressed the ox-LDL- or TM-induced activation of ER stress signaling pathway including the phosphorylation of double-stranded RNA-activated protein kinase-like ER kinase (PERK) and eukaryotic translation initiation factor 2α (eIF2α) as well as upregulation of glucose regulated protein 78 (GRP78) and the pro-apoptotic protein CHOP. Furthermore, EEP significantly suppressed ox-LDL intake by macrophages and the upregulation of CD36 induced by ox-LDL.

**Conclusion:**

These data indicate that EEP may protect macrophages from ox-LDL-induced apoptosis and the mechanism at least partially involves its ability to suppress the CD36-mediated ox-LDL intake and subsequent activation of ER stress-CHOP signalling pathway.

## Background

Apoptosis, especially in macrophages-dense atherosclerotic lesions, is unanimously considered as a prominent feature of advanced atherosclerotic plaques, suggesting that macrophage apoptosis is closely related to the atherosclerotic development and subsequent plaque rupture, which is the prominent event that results in the majority of clinical manifestations of acute coronary syndrome such as acute myocardial infarction and sudden coronary death [1]. Macrophage apoptosis at early stages, combined with efficient clearance of apoptotic cells (efferocytosis), helps in maintaining reduced cellularity and slows lesion progression. However in advanced atherosclerotic lesions where efferocytosis is defective, apoptosis of macrophage- derived foam cells contributes to the formation and expansion of lipid core, and gives rise to inflammation and necrosis, which lead to plaque instability [2]. Thus, protecting macrophages from apoptosis is believed as an effective approach to attenuate plaque instability and combat acute vascular events [3].

Propolis, a resinous hive product collected by honeybees from many plant sources, contains predominantly phenolic compounds including flavonoids, phenolic acids and their esters [4–6]. Propolis has a long history of being used in traditional medicine because of its broad spectrum of biological activities incoluding anticancer, antioxidant, antiinflammatory, antibiotic and antifungal properties [7]. Recent studies have shown that ethanol extract of propolis (EEP), rich in flavones, has inhibitory effects on inflammatory responses and inflammation-related transcription factors in macrophages [8, 9]. In our previous studies, we have found that EEP promotes reverse cholesterol transport and restrians atherosclerotic lesion development [10, 11]. More interestingly, our previous studies have shown that EEP protects endothelial cells from oxidized low density lipoprotein (ox-LDL)-induced injury by inhibiting lectin-like oxidized low density lipoprotein receptor-1 (LOX-1)-mediated oxidative stress [12]. However, whether EEP protects macrophages from ox-LDL-induced apoptosis by inhibiting endoplasmic reticulum (ER) stress-C/EBP homologous protein (CHOP) pathway has not yet been determined.

CHOP, belonging to C/EBP transcription factor family, is a specific transcription factor in ER stress [13]. ER stress-CHOP pathway-mediated apoptosis in macrophages has been confirmed to promote the instability of atherosclerotic plaques, and the deficiency of CHOP has been discovered to prevent macrophages from ER stress-induced apoptosis *in vitro* and in advanced atherosclerotic lesions of mice [14, 15]. Therefore, a positive correlation among CHOP expression, apoptosis of macrophages and progression of atherosclerotic plaques to the vulnerable stage is undoubted [2, 15, 16]. We have previously reported that both minimally modified LDL (mm-LDL) and ox-LDL can induce ER stress during the formation of macrophage-derived foam cells, and double-stranded RNA-activated protein kinase-like ER kinase (PERK) mediates ox-LDL-induced macrophage apoptosis by up-regulating CHOP expression [17, 18]. In addition, our earlier study has provided preliminary evidence that quercetin, one of the flavonoids that are ubiquitous in plants and propolis, protects macrophages from ox-LDL-induced apoptosis by inhibiting CHOP expression [19]. Therefore, we hypothesize that EEP may protect macrophages from ox-LDL-induced apoptosis through suppressing ER stress-CHOP signalling pathway. In this present study, we explored the protective effect of EEP on ox-LDL-induced cytotoxicity in RAW264.7 macrophages and specifically the ER stress-CHOP pathway-mediated apoptosis.

## Methods

### Reagents

Tunicamycin (TM), oil red O, 4-phenylbutyric acid (PBA) and rabbit antibody against β-actin were purchased from Sigma-Aldrich (St Louis, MO, USA). Dulbecco’s modified Eagle medium (DMEM) and fetal bovine serum (FBS) were obtained from Gibco (Rockville, MD, USA). RIPA lysis buffer and DiI-ox-LDL were from Solarbio (Beijing, China) and Xiesheng Biotech (Beijing, China), respectively. Rabbit polyclonal antibodies against glucose regulated proteins 78 (GRP78), CHOP, double-stranded RNA-activated protein kinase-like ER kinase (PERK), eukaryotic translation initiation factor 2α (eIF2α) and phospho-eIF2α (p-eIF2α) were purchased from Santa Cruz Biotechnology (Santa Cruz, CA, USA). Rabbit antibodies against phospho-PERK (p-PERK) and anti-CD36 monoclonal antibody (mAb) were purchased from Abcam (Cambridge, MA, USA). SABC-Cy3 immunohistochemistry kits were obtained from Boshide (Wuhan, China). Annexin V-FITC apoptosis detection kits, 3-(4,5-dimethylthiazol- 2-y-l)-2,5-diphenyl-2H-tetrazolium bromide (MTT) and lactate dehydrogenase (LDH) assay kits were obtained from BD Biosciences (San Jose, CA, USA), Genview (Houston, TX, USA) and Jiancheng Biotech (Nanjing,China), respectively. Enhanced chemiluminescence (ECL) kits and polyvinylidene fluoride (PVDF) membranes were obtained from Thermo Scientific Pierce (Rockford, IL, USA) and Millipore (Bedford, MA, USA), respectively. Caspase-3 activity assay kit and terminal deoxynucleotidyl transferase-mediated dUTP nick end-labeling (TUNEL) assay kit were from Calbiochem (San Diego, CA, USA) and Roche (Mannheim, Germany), respectively. Real-time PCR reagent kits were purchased from Tiangen Biological Chemistry (Beijing, China). Tissue/cell total cholesterol (TC) assay kits were obtained from Applygen (Beijing, China).

### Preparation of EEP and total flavonoids measurement

Propolis was harvested from Taishan Mountain in Shandong province (China) and EEP was prepared as described in our recent report [12]. Briefly, propolis powder (100 g) was extracted in 95 % (v/v) ethanol (1 L) under the condition of sonication at 40 °C for three times, and then the supernatant were evaporated in a rotary evaporator under a reduced pressure at 50 °C. Finally, the EEP was dried in the oven and stored at -20 °C. The total flavonoids content of EEP was 213.46 ± 2.93 mg rutin equivalent per gram according to the colorimetric method of Chinese Standard (GB/T 20574-2006). Immediately prior to use, EEP sample was dissolved in dimethylsulfoxide (DMSO) and diluted with cell culture medium into appropriate concentrations.

### Isolation and oxidation of LDL

Human LDL was isolated from fresh plasma of healthy donors using sequential ultracentrifugation, and then oxidized with 10 μmol/L CuSO_4_ for 18 h at 37 °C as described in our previous study [17]. The use of human blood and protocol of human LDL isolation were approved by the Ethics Committee of Taishan Medical University, and all blood donors provided informed consent for the use of their blood.

### Cell culture

RAW264.7 macrophages were purchased from the Type Culture Collection of the Chinese Academy of Sciences (Shanghai, China), cultured in DMEM supplemented with 2 mM glutamine, antibiotics (100 U/ml penicillin and streptomycin) and 10 % FBS in a 37 °C humidified incubator containing 5 % CO_2_ until subconfluent, and then the medium was replaced with serum-free medium for 12 h before treatment.

### Oil red O staining

The intracellular lipid droplets were stained by oil red O and the lipid droplet content was expressed as the average value of the integrated optical density (IOD) as described previously [17].

### Intracellular TC analysis

The intracellular TC concentration was measured using a tissue/cell TC assay kit according to the manufacturer’s instructions and normalized to the level of total cellular protein.

### Cell viability and LDH assay

The viability of the treated cells grown in 96-well plates was evaluated using MTT assay as described previously [17] and cell viability was expressed as the percentage of the control group.

The release of the cytosolic LDH into the medium was used as a generic index of cell injury. After treatment, the media were collected and assayed for LDH activity using a LDH activity assay kit according to the manufacturer’s instructions.

### Flow cytometry analysis of apoptotic cells

The Annexin V-FITC/PI double-staining assay was used to quantify apoptosis. Cells of each group were collected, washed with ice-cold PBS twice and centrifuged at 4 °C. 500 μL binding buffer was added to resuspend the cells, then 5 μL Annexin V-FITC and 5 μL PI were added. The cells were incubated for 15 min in the dark at room temperature, and the apoptosis rates were analyzed on a FACScan flow cytometer using Cell Quest software (Becton Dickinson, San Jose, CA, USA).

### TUNEL staining

DNA strand breaks in the treated macrophages were detected using the TUNEL staining kit according to the manufacturer’s instructions. Cells were fixed with 4 % paraformaldehyde for 30 min at room temperature, rinsed with PBS, and then incubated in 0.1 % triton X-100 permeabilisation solution for 2 min on ice. The TUNEL reaction mixture was added to the cells and incubated for 1 h in a dark humidified chamber at 37 °C. After incubation, the cells were washed twice for 5 min in PBS and stained with 4’,6-diamidino-2’-phenylindole dihydrochloride (DAPI). Cells were detected using a fluorescence microscope (Olympus, Tokyo, Japan) and the ratio of TUNEL-positive cells to total cells was calculated.

### Measurement of caspase-3 activity

Caspase-3 activity was identified with an assay kit according to the manufacturers’ instructions. Briefly, following treatment, RAW264.7 cells were harvested, rinsed with PBS and lysed with lysis buffer. The lysate was centrifuged at 12000 g at 4 °C for 15 min. 20 μl lysate supernatant was added to 70 μl reaction buffer and 10 μl caspase-3 substrate, and then the mixture was incubated in 96-well microtiter plates at 37 °C for 2 h. Caspase-3 activity was detected by an Infinite F200 microplate reader (Tecan, Switzerland) at 405 nm and described as a percentage of the control.

### Western blot analysis

At the end of treatment, the cells were washed and lysed in RIPA buffer. Equal amounts of protein were separated on SDS-PAGE by electrophoresis and then transferred onto a PVDF membrane. After blocking in 10 % nonfat dry milk for 2 h at room temperature, the membranes were probed with primary antibodies overnight at 4 °C and then incubated with horseradish peroxidase-conjugated secondary antibodies for 1 h at room temperature. The bound immunoproteins were visualized by ECL reaction, and then the intensities were quantified by Image-Pro Plus software (version 6.0, Media Cybernetics, LP, USA) and normalized to β-actin levels.

### Quantitative real time-PCR analysis

Toal RNA from the treated cells were isolated using Trizol reagent (Invitrogen), and synthesized to cDNA using MuLV Reverse Transcriptase. Real-time PCR were performed on a Rotor-Gene Q real-time PCR cycler (Qiagen, Shanghai, China) using SYBR-green PCR master mix kits as described previously [17, 19]. The primers were synthesized by Sangon Biotech (Shanghai, China) and the oligonucleotide sequences were as follows: CHOP: 5’-CCACCACACCTGAAAGCAGAA-3’(forward primer), 5’-GGTGCCCCCAATTTCATCT-3’(reverse primer); GRP78: 5’-ACATGGACCTG TTCCGCTCTA-3’ (forward primer), 5’-TGGCTCCTTGCCATTGAAGA-3’ (reverse primer); β-actin: ’-CGGGGACCTGACTGACTACC-3’ (forward primer), 5’-AGGA AGGCTGGAAGAGTGC-3’ (reverse primer). The data were analyzed using the Rotor- Gene Q software (version 1.7, Qiagen), and then relative mRNA levels were calculated by the 2^–△△Ct^ method.

### Uptake of Dil-ox-LDL

Cells were pretreated with EEP (7.5, 15 and 30 mg/L) or anti-CD36 mAb (2 mg/L) for 1 h and then incubated with Dil-ox-LDL (50 mg/L) for 6 h. Cells were washed with PBS and lysed in 200 μL lysis buffer. Using an Infinite F200 microplate reader (Tecan, Switzerland), the levels of Dil-ox-LDL in cell lysate were quantitated as reported previously [20], and these data were standardized to the protein concentration of each sample.

The uptake of Dil-ox-LDL by RAW264.7 cells was further assayed by fluorescence microscopy. Cells were washed with PBS, fixed with 4 % paraformaldehyde for 20 min, and the cell nuclei was counterstained with DAPI for 20 min. After washing for three times, the cells were observed using a fluorescence microscope (Olympus, Tokyo, Japan) and the mean fluorescence intensity per cell was calculated using Image-Pro Plus software 6.0.

### Immunofluorescence assay for CD36 expression

The treated cells were fixed with 4 % (w/v) paraformaldehyde, blocked with BSA, and then incubated with CD36 antibody (1:100) overnight at 4 °C. After incubation with secondary FITC-conjugated antibody and DAPI, the cells were washed with PBS, mounted in antifade reagent and then observed using an Olympus BX51 microscopes as described previously [20].

### Statistical analysis

Results are expressed as the mean ± SEM. Statistical analysis was performed by one-way analysis of variance with Student–Newmann–Keuls multiple comparison tests using the SPSS13.0 software for Windows. *P-*values less than 0.05 were considered significant.

## Results

### EEP suppresses ox-LDL-induced lipid cumulation in RAW264.7 cells

**Fig. 1:**
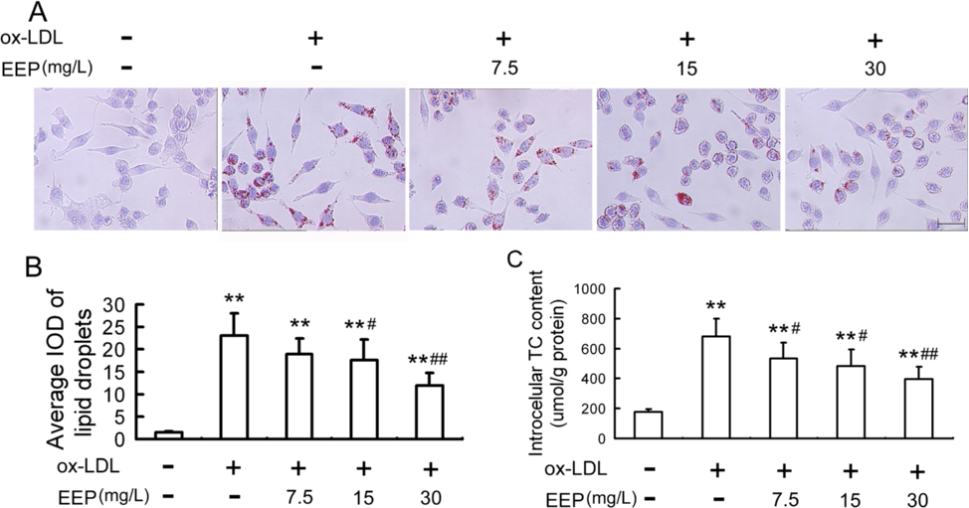
EEP reduces ox-LDL-induced intracellular lipid accumulation in RAW264.7 cells. **a** Cells were treated with ox-LDL (100 mg/L) in the absence or presence of EEP at different concentrations (7.5, 15 and 30 mg/L) for 24 h, and the intracellular lipid droplets were stained by oil red O. Representative lipid droplet staining images are shown. Scale bar = 20 μm. **b** The average integrated optical density (IOD) of lipid droplets stained with oil red O from differentiated macrophage-derived foam cells was calculated. **c** Under the same conditions as in (**a**), the intracellular total cholesterol (TC) content was measured using a tissue/cell TC assay kit. Data are expressed as the mean ± SEM of at least four independent experiments. **P < 0.01 versus vehicle-treated control; ^#^ *P* < 0.05, ^##^ *P* < 0.01 versus ox-LDL treatment

Lipid droplets stained by oil red O were observed significantly in RAW264.7 cells treated with 100 mg/L of ox-LDL for 24 h. However, the ox-LDL-induced cumulation of lipid droplets was reduced by EEP in a dose-dependent manner (Fig. 1a and b). Lipid cumulation was further certified by intracellular TC quantitative assay. Compared with the control, intracellular TC content was remarkably increased (3.9-fold) by ox-LDL treatment. However, the effect of ox-LDL was attenuated by 21.3 %, 28.6 % and 41.3 % upon EEP treatment at 7.5, 15 and 30 mg/L, respectively (Fig. 1c).

### EEP attenuates cytotoxicity in RAW264.7 cells induced by ox-LDL or TM

**Fig. 2:**
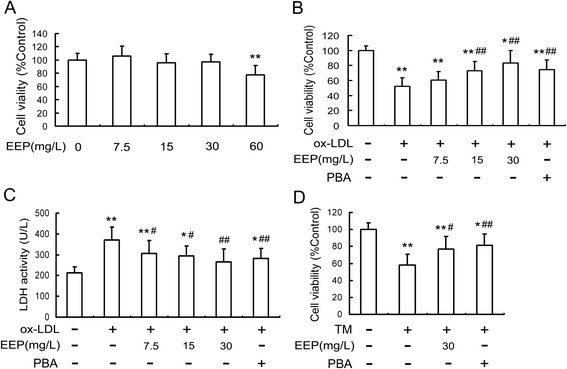
Effects of EEP on ox-LDL- or TM-induced cytotoxicity in RAW264.7 cells. **a** Determination of the cytotoxic effects of EEP on RAW264.7 cells. Cells were incubated with the indicated concentrations of EEP for 24 h and cell viability was measured by the MTT assay and expressed as the percentage of control. **b** and **c** EEP inhibits ox-LDL-induced cell death and LDH leakage. RAW264.7 cells were pretreated with EEP (7.5, 15 and 30 mg/L) or PBA (5 mmol/L) for 1 h, and then exposed to ox-LDL (100 mg/L) for 24 h. **d** EEP inhibits TM-induced cell death. Cells were pretreated with EEP (30 mg/L) or PBA (5 mmol/L) for 1 h, and then incubated with TM (5 mg/L) for 12 h. Data are expressed as the mean ± SEM of six independent experiments. *P < 0.05, **P < 0.01 versus vehicle-treated control; ^#^ *P* < 0.05, ^##^ *P* < 0.01 versus ox-LDL or TM treatment

Cytotoxicity of EEP on RAW264.7 cells was assessed by MTT assay. Except for the concentration of 60 mg/L, stimulation with EEP at 7.5 up to 30 mg/L for 24 h had no remarkable effect on cell viability (Fig. 2a). Next, we determined the protective effect of EEP on ox-LDL-induced cell death. The treatment with 100 mg/L ox-LDL for 24 h reduced cell viability by approximately 47.7 % as measured using MTT assay. However, preincubation with different doses of EEP (7.5, 15 and 30 mg/L) increased the cell viability in a dose-dependent manner compared with cells treated with ox-LDL (Fig. 2b).

LDH, which leaks from cells after plasma membrane injury, was determined to further certify the protective effect of EEP on RAW264.7 cells. As shown in Fig. 2c, LDH release increased dramaticly in the media after cells were incubated with ox-LDL. However, pretreatment with EEP dramaticly reduced the LDH release in a dose-dependent manner.

To determine the protective effect of EEP on ER stress-mediated cell death, PBA, an ER stress inhibitor, was used as a positive control. As shown in Fig. 2b and c, PBA also prevented the decreased cell viability and the LDH release induced by ox-LDL. In addition, TM, an ER stress inducer by inhibiting protein glycosylation, was also used in the study to develop ER stress model. When cells were preincubated with EEP (30 mg/L) before TM (5 mg/L) treatement for 12 h, the cell viability raised by 32.5 % compared with TM exposure alone, which was similar to PBA pretreatment (Fig. 2d). These data indicated that EEP was able to restrain ER stress-induced cell death.

### EEP attenuates apoptosis in RAW264.7 cells induced by ox-LDL or TM

**Fig. 3:**
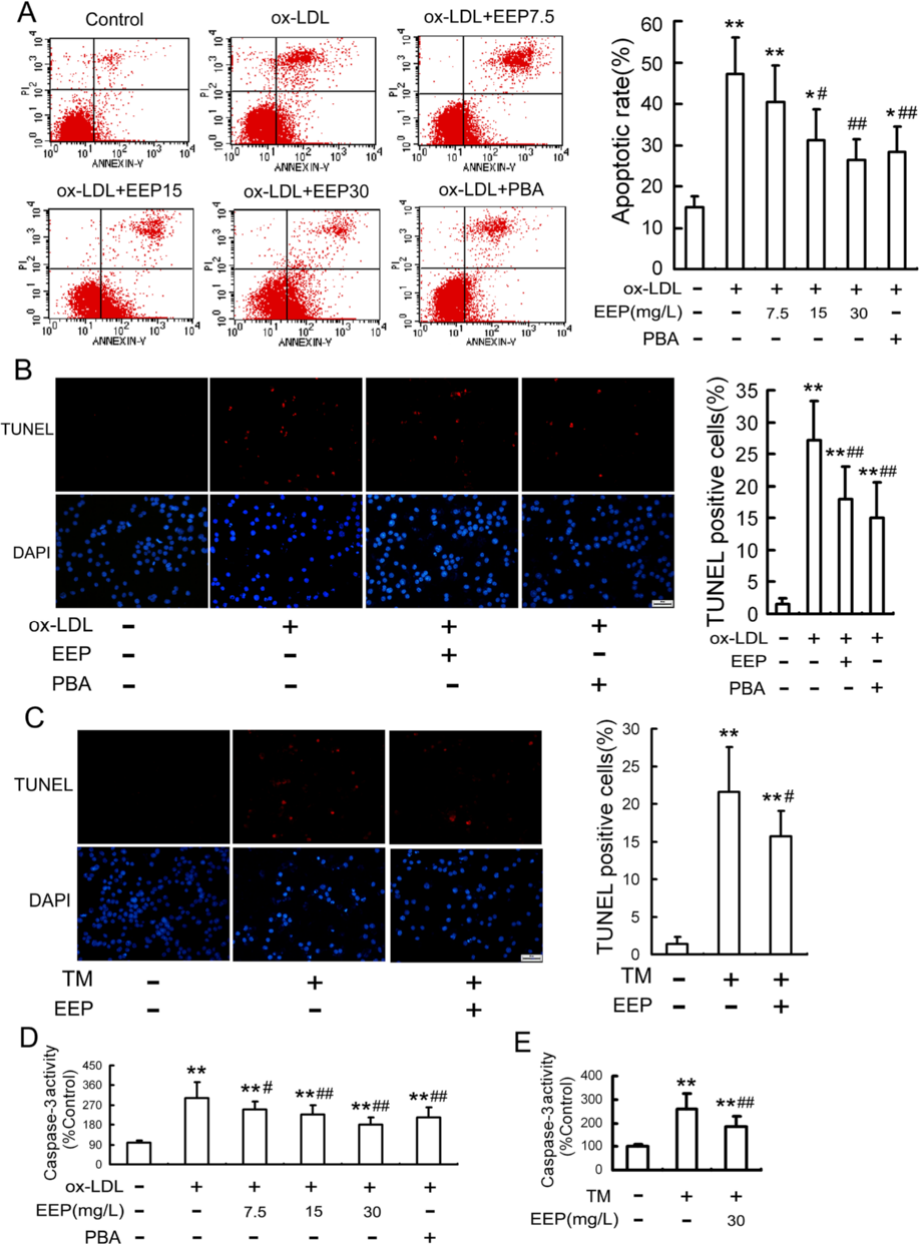
Effect of EEP on ox-LDL- or TM-induced apoptosis in RAW264.7 cells. **a** After RAW264.7 cells were treated with EEP (7.5, 15 and 30 mg/L) or PBA (5 mmol/L) in the presence of ox-LDL (100 mg/L) treatment for 24 h, cell apoptosis was detected using flow cytometry and the total apoptotic cells (early and late-stage apoptosis) were represented by the right side of the panel (Annexin V staining alone or together with PI). **b** and **c** Cells were pretreated with EEP (30 mg/L) or PBA (5 mmol/L) for 1 h, followed by exposure to ox-LDL (100 mg/L) for 24 h or TM (5 mg/L) for 12 h, and then cell apoptosis was detected by TUNEL assay. Scale bar =20 μm. **d** and **e** Cells were pretreated with the indicated concentrations of EEP or PBA (5 mmol/L) in the presence of ox-LDL (100 mg/L) for 24 h or TM (5 mg/L) treatment for 12 h, and then caspase-3 activity was determined by colorimetric assay. Data are expressed as the mean ± SEM of at least four independent experiments. *P < 0.05, **P < 0.01 versus vehicle-treated control; ^#^ *P* < 0.05, ^##^ *P* < 0.01 versus ox-LDL or TM treatment

To investigate whether EEP could attenuate ox-LDL-induced apoptosis, the apoptotic rate of the treated cells were analyzed by flow cytometry. RAW264.7 cells were induced apoptosis with ox-LDL (100 mg/L) for 24 h, whereas EEP pretreatment (7.5, 15 and 30 mg/L) attenuated ox-LDL-induced apoptosis in a dose-dependent manner. Similarly, the inhibitory effect of PBA on ox-LDL-induced apoptosis was also observed (Fig. 3a).

TUNEL staining was performed to further confirm the anti-apoptotic effects of EEP. Similar results were observed using cells pretreated with 30 mg/L EEP or 5 mmol/L PBA compared with ox-LDL treatment alone (Fig. 3b). Additionally, TUNEL analysis showed that the increase in apoptotic rate induced by TM was attenuated by the addition of EEP (Fig. 3c).

The activity of caspase-3, a marker of apoptosis, was also determined in our study. As illustrated in Fig. 3d and e, the ox-LDL- or TM-induced increase in caspase-3 activity was remarkably inhibited by EEP in a dose-dependent manner. Moreover, the ox-LDL-induced activation of caspase-3 was also inhibited by PBA (Fig. 3d).

### EEP inhibits ox-LDL-induced ER stress response in RAW264.7 cells

**Fig. 4:**
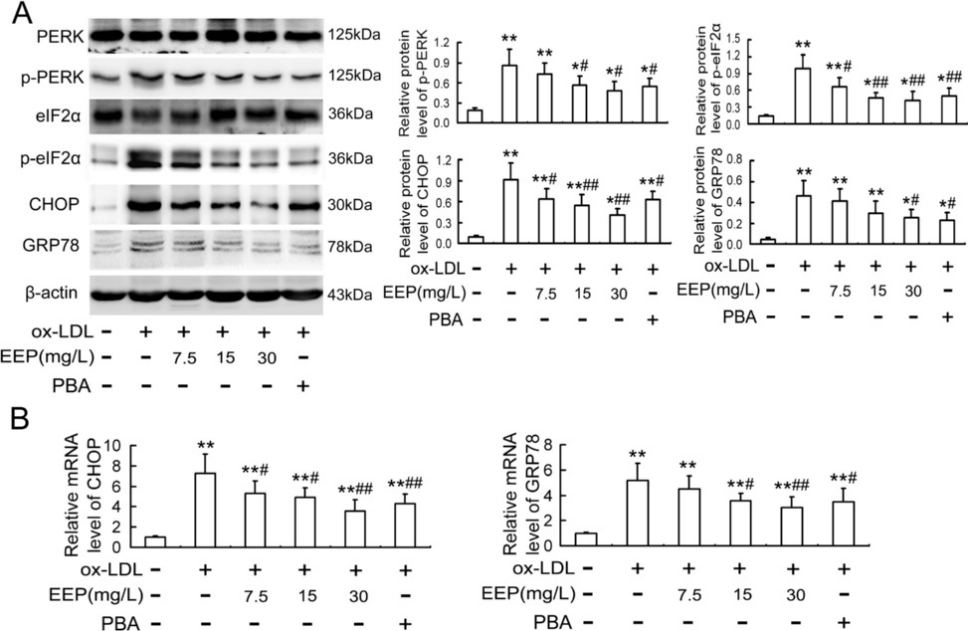
EEP inhibits ox-LDL-induced ER stress response in RAW264.7 cells. Cells were pretreated with the indicated concentrations of EEP or PBA (5 mmol/L) in the presence of ox-LDL (100 mg/L) for 24 h, and then the protein (**a**) and mRNA (**b**) levels of ER stress markers were evaluated by Western blot and quantitative real-time PCR, respectively. Data are expressed as the mean ± SEM of at least three independent experiments. *P < 0.05, **P < 0.01 versus vehicle-treated control; ^#^ *P* < 0.05, ^##^ *P* < 0.01 versus ox-LDL treatment

CHOP is involved in the process of apoptosis associated with ER stress, while PERK is one of important upstream molecules that play an important role in the induction of CHOP. ^20^ Since we confirmed that like PBA, EEP could attenuate the ox-LDL- and TM-induced apoptosis in RAW264.7 cells (Fig. 3), we next examined the effect of EEP on ER stress-CHOP pathway. As seen in Fig. 4 a, ox-LDL caused significantly the protein upregulation of p-PERK, p-eIF2α, GRP78 and CHOP in RAW264.7 cells compared with the control cells, which were remarkably inhibited by EEP in a dose-dependent manner. Consistent with the Western blot results, ox-LDL-induced upregulation of CHOP and GRP78 mRNA were also attenuated by EEP (Fig. 4b). Similar results were obtained using the cells pretreated with PBA (Fig. 4a and b).

### EEP inhibits TM-induced ER stress response in RAW264.7 cells

**Fig. 5:**
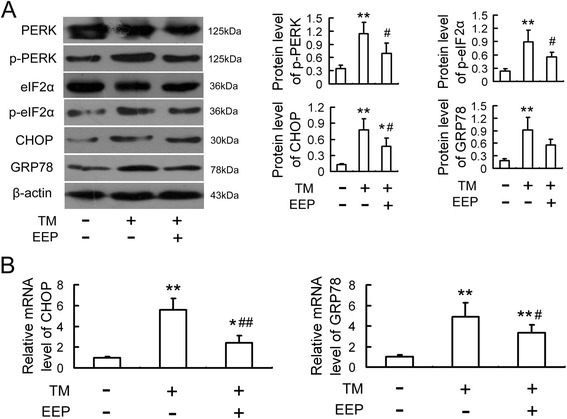
EEP inhibits TM-induced ER stress response in RAW264.7 cells. Cells were pretreated with EEP (30 mg/L) in the presence of TM (5 mg/L) treatment for 12 h, and then the protein (**a**) and mRNA (**b**) levels of ER stress markers were evaluated by Western blot and quantitative real-time PCR, respectively. Data are expressed as the mean ± SEM of at least three independent experiments. *P < 0.05, **P < 0.01 versus vehicle-treated control; ^#^ *P* < 0.05, ^##^ *P* < 0.01 versus TM treatment

To further confirm EEP could reduce the apoptosis of RAW264.7 cells by inhibiting ER stress-CHOP pathway, we explored the changes of ER stress markers on TM-induced ER stress model. Similar to the results above, pretreatment with EEP could inhibit TM-induced phosphorylation of PERK and eIF2α and the upregulation of GRP 78 and CHOP (Fig. 5a and b).

### EEP suppresses ox-LDL uptake and CD36 expression in RAW264.7 cells

**Fig. 6:**
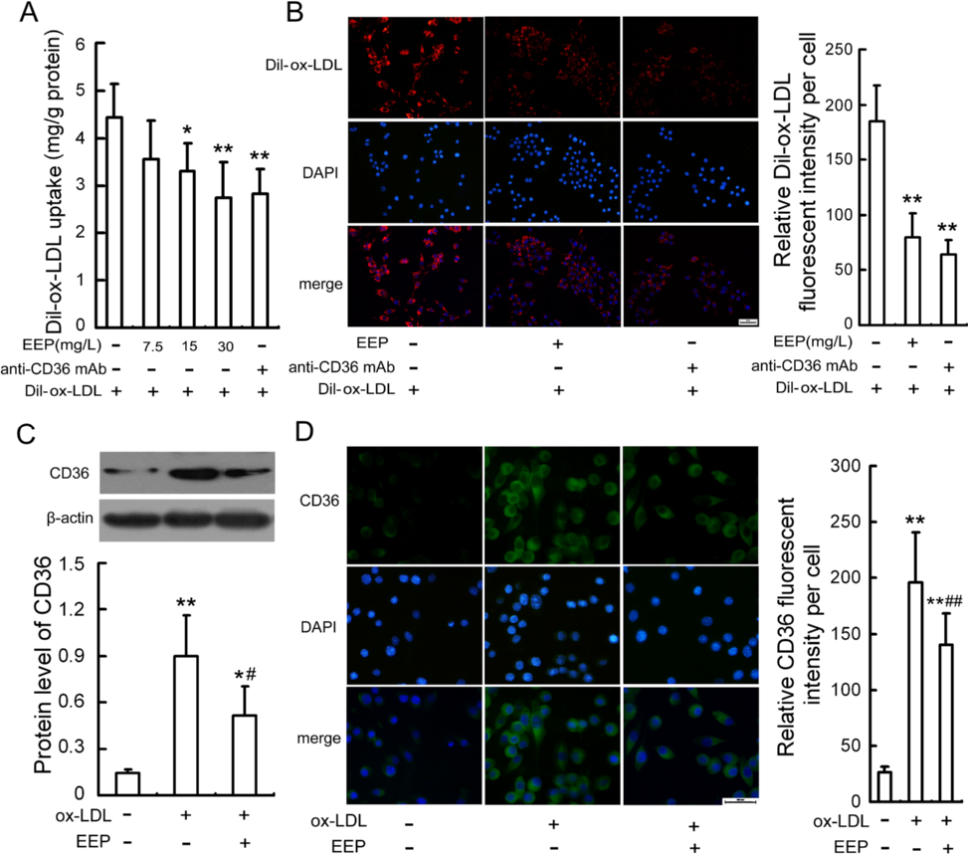
EEP attenuates ox-LDL uptake and CD36 upregulation in RAW264.7 cells. **a** Dil-ox-LDL fluorescence intensity in cells preincubated with EEP at the indicated concentrations or anti-CD36 mAb (2 mg/L) for 1 h and then treated with Dil-ox-LDL (50 mg/L) for 6 h. **b** Fluorescence microscopy showed Dil-ox-LDL uptake by RAW264.7 cells preincubated with EEP (30 mg/L) or anti-CD36 mAb (2 mg/L) for 1 h and then treated with Dil-ox-LDL (50 mg/L) for 6 h. Red staining denotes Dil-ox-LDL fluorescence and the blue staining denotes nuclei visualized by DAPI (Scale bar = 20 μm). **c** and **d** Protein expression of CD36 in cells pretreated with EEP (30 mg/L) in the presence of ox-LDL (100 mg/L) for 24 h were evaluated by Western blot and immunofluorescence assay, respectively. Representative fluorescent images are shown. Green, CD36 visualized by FITC labeling; blue, nuclei visualized by DAPI. Scale bar = 20 μm. Data are expressed as the mean ± SEM of at least three independent experiments. *P < 0.05, **P < 0.01 versus vehicle-treated control; ^#^ *P* < 0.05, ^##^ *P* < 0.01 versus ox-LDL treatment

Since about 60 %–70 % of macrophage-derived foam cell formation is caused by CD36-mediated ox-LDL uptake, which is an important factor to lead to ER stress [20, 21], and EEP suppressed ox-LDL-induced lipid cumulation in RAW264.7 cells (Fig. 1), we next detected whether the mechanism underlying the regulatory effect of EEP on ER stress-CHOP pathway could be through inhibition of CD36 expression. Our results demostrated that the uptake of Dil-ox-LDL in RAW264.7 cells was reduced by EEP in a concentration-dependent manner, which was similar to anti-CD36 mAb treatment (Fig. 6a and b). In addition, Western blot and immunofluorescence results (Fig. 6c and d) showed that ox-LDL-induced CD36 upregulation was significantly restrained by EEP, suggesting that EEP may inhibit the uptake of ox-LDL via supression of CD36 expression.

## Discussion

Apoptosis of lipid-containing macrophages in the advanced atherosclerotic lesion promotes inflammatory response, lesional necrosis and enlargement of the lipid core, which lead to plaque rupture and acute thrombosis [1, 2]. Thus, inhibition of macrophage apoptosis may be effective in blocking acute cardiovascular events. In the present study, we first demonstrated that EEP not only inhibited ox-LDL-induced macrophage-derived foam cell formation, injury and apoptosis but also reduced ER stress inducer TM-induced macrophage injury and apoptosis, which were similar to PBA (an ER stress inhibitor) pretreatment. Second, like PBA, EEP suppressed ox-LDL or TM-induced ER stress-CHOP pathway via inhibiting PERK activation. In addition, EEP mitigated ox-LDL uptake by macrophages and inhibited CD36 upregulation induced by ox-LDL. These data indicate that EEP inhibits ox-LDL-induced macrophage apoptosis by suppressing CD36-mediated ox-LDL uptake and subsequent activation of ER stress-CHOP pathway.

In recent years, natural medicine especially the plant flavonoids with cardiovascular protective function gained much attention and were proven to reduce the risk of cardiovascular desease *in vitro* and *in vivo* based on different animal models [22]. Icariin (a prenylated flavonol glycoside) attenuates cardiac remodelling through down-regulating myocardial apoptosis in rats with congestive heart failure [23]. Wogonin suppresses myocardial apoptosis induced by ischemia/reperfusion in rats [24]. Quercetin (a member of the bioflavonoid family) is confirmed to exhibit antiapoptotic properties in various cell types [25–27]. Acumulating evidence has demonstrated that EEP and its flavones exhibit anti-inflammatory effects in macrophages [8, 9]. In addition, our previous studies have revealed that EEP promotes reverse cholesterol transport, protects endothelial cells from ox-LDL-induced apoptosis and inhibits atherosclerotic lesion development [10–12]. However, whether EEP could inhibit ox-LDL-induced macrophage apoptosis remains unkown. Our results in the present work showed that EEP remarkably restrained ox-LDL-induced lipid cumulation and macrophage insult as reflected by the increased cell viability and the decreased LDH leakage, caspase-3 activation and apoptosis, suggesting that EEP is able to attenuate ox-LDL-induced macrophage-derived foam cell injury.

ER is a critical cell organelle which takes part in cell accommodation and death [28]. Accumulating data have indicated that ER stress-mediated apoptosis is associated with plaque instability in atherosclerosis and its vascular complications [16]. ER stress is well known to activate the unfolded protein response (UPR) as an adaptive response to the disequilibrium in ER homeostasis through transient translational inhibition, upregulation of ER molecular chaperone and activation of ER-associated degradation (ERAD). However, when such correction is not satisfactorily achieved due to severe or prolonged ER stress, the pro-apoptotic signaling pathway will be elicited. One of the important signaling mediators in the ER stress-mediated apoptosis pathway is CHOP [15, 29]. Numerous studies have demonstrated that CHOP is markedly elevated and contributes to macrophage apoptosis and the instability of atherosclerotic plaques, whereas CHOP deficiency attenuates macrophage apoptosis and atherosclerotic plaque necrosis, suggesting that activation of CHOP is the key signaling step in macrophage apoptosis and plaque instability [14, 15, 30]. PERK is one of upstream molecules that play a critical role in the induction of CHOP in ER stress. PERK is type I ER transmembrane protein possessing serine/threonine kinase activity. Activated PERK in response to ER stress induces phosphorylation of eIF2α, thereby reducing the overall protein translational levels, which attenuates the unfolded protein load in ER. In addition, eIF2α phosphorylation also facilitates the expression of the activating transcription factor 4 (ATF4) that translocates into the nucleus and increases CHOP expression [16]. Our recent work showed that PERK mediated ox-LDL-induced apoptosis in macrophages by up-regulating CHOP expression [17]. In the present study, we discovered that EEP downregulated CHOP expression as well as inhibiting the phosphorylation of PERK and eIF2α induced by ox-LDL in RAW264.7 cells, which was similar to the ER stress inhibitor PBA. TM has been known to facilitate apoptosis by activating ER stress signaling pathways [30]. To further confirm the regulatory effect of EEP on ER stress-CHOP pathways, the TM-induced macrophage apoptosis model was used in the present study. Our data revealed that EEP reduced the TM-induced cytotoxicity and upregulation of CHOP. The phosphorylation of PERK and eIF2α induced by TM were also dramatically inhibited by EEP. These results indicate that the inhibition of ox-LDL-induced apoptosis by EEP is related to the repression of ER stress-CHOP pathway.

Several observations including our previous studies have demonstrated that the cumulation of intracellular cholesterol is a crucial inducer of ER stress and macrophage apotosis *in vivo* and *in vitro* [17, 18, 31, 32]. CD36, a class B scavenger receptor that is expressed on a wide variety of cells especially on monocytes/macrophages, has been identified as the major receptor responsible for ox-LDL uptake into macrophages and cholesterol accumulation [33, 34]. Loss or inhibition of CD36 resultes in significant decreased ability of macrophages to accumulate ox-LDL and cholesteryl, and protectes against atherosclerotic lesion development [35–37]. These data point to a significant role of CD36 in atherosclerosis development and suggest it could be an important target for therapeutic treatment. More interestingly, our recent study has provided that CD36 silencing attenuates ox-LDL-induced ATF6 nuclear translocation and GRP78 upregulation, suggesting that CD36-mediated ox-LDL uptake in macrophages triggers ER stress response [20]. Our present work showed that similar to anti-CD36 antibody, EEP significantly suppressed ox-LDL intake as well as the upregulation of CD36 induced by ox-LDL, which may be the mechanism for the inhibitory effect of EEP on the ER stress-CHOP pathway-mediated macrophage apoptosis induced by ox-LDL.

## Conclusions

The present study showed for the first time that EEP protected macrophages from ox-LDL-induced apoptosis through the inhibition of the CD36-mediated ox-LDL intake and subsequent activation of ER stress-CHOP signalling pathway *in vitro*. The results might explain the diverse physiological activities of EEP and emphasize the pharmacological application of EEP in atherosclerosis-related diseases.

## Abbreviations

EEP: Ethanol extract of propolis
ox-LDL: oxidized low-density lipoprotein
ER: Endoplasmic reticulum
TC: Total cholesterol
CHOP: C/EBP homologous protein
TM: Tunicamycin
PBA: 4-phenylbutyric acid
PERK: RNA- activated protein kinase-like ER kinase
eIF2α: eukaryotic translation initiation factor 2α
GRP78: Glucose regulated protein 78
LOX-1: Lectin-like oxidized low density lipoprotein receptor-1
mm-LDL: minimally modified LDL
p-eIF2α: phospho- eukaryotic translation initiation factor 2α
p-PERK: phospho-double- stranded RNA- activated protein kinase-like ER kinase
DMSO: Dimethylsulfoxide
IOD: Integrated optical density
LDH: dehydrogenase
MTT: 3-(4,5- dimethylthiazol- 2-y-l)- 2,5- diphenyl-2H-tetrazolium bromide
TUNEL: Terminal deoxynucleotidyl transferase- mediated dUTP nick end-labeling
ATF4: Activating transcription factor 4
DAPI: 4’, 6-diamidino-2-phenylindole dihydrochloride
